# Epstein-Barr Virus BART Long Non-coding RNAs Function as Epigenetic Modulators in Nasopharyngeal Carcinoma

**DOI:** 10.3389/fonc.2019.01120

**Published:** 2019-10-22

**Authors:** Rob J. A. Verhoeven, Shuang Tong, Bobo Wing-Yee Mok, Jiayan Liu, Songtao He, Jingfeng Zong, Yixin Chen, Sai-Wah Tsao, Maria Li Lung, Honglin Chen

**Affiliations:** ^1^State Key Laboratory for Emerging Infectious Diseases, Department of Microbiology and the Collaborative Innovation Center for Diagnosis and Treatment of Infectious Diseases, Li Ka Shing Faculty of Medicine, The University of Hong Kong, Hong Kong, China; ^2^Department of Radiation Oncology, Fujian Provincial Cancer Hospital, Fuzhou, China; ^3^School of Life Sciences, National Institute of Diagnostics and Vaccine Development in Infectious Diseases, Xiamen University, Xiamen, China; ^4^Li Ka Shing Faculty of Medicine, School of Biomedical Sciences, The University of Hong Kong, Hong Kong, China; ^5^Department of Clinical Oncology, Li Ka Shing Faculty of Medicine, The University of Hong Kong, Hong Kong, China

**Keywords:** Epstein-Barr virus, nasopharyngeal carcinoma, lncRNA, BART, epigenetics, Aiolos

## Abstract

Epstein-Barr virus (EBV) establishes lifelong latent infection in humans and is associated with several lymphoid and epithelial cancers. In nasopharyngeal carcinoma (NPC), EBV expresses few viral proteins but elevated levels of *Bam-*HI A rightward transcripts (BARTs) RNA, which includes viral microRNAs and long non-coding RNAs (lncRNAs). BART lncRNAs localize within the nucleus of EBV-infected cells and knockdown of BART lncRNAs significantly affects the expression of genes associated with cell adhesion, oxidoreductase activity, inflammation, and immunity. Notably, downregulation of *IKAROS* family zinc finger 3 (*IKZF3/*Aiolos), which plays a role in lymphocyte development and cell attachment, occurred in NPC C666-1 cells following BART lncRNA-knockdown. Since Aiolos expression is normally restricted to lymphoid cells and rarely observed in epithelial cells, induction of Aiolos by BART lncRNA was confirmed by expressing the major BART lncRNA isoform, *RPMS1*, in EBV-positive and -negative cells. BART lncRNA associated with the CBP/p300 complex and RNA polymerase II (Pol II) in the nucleus, suggesting that BART lncRNAs may mediate epigenetic regulation of gene expression through interaction with the chromatin remodeling machinery. This contention is further supported by evidence that BART lncRNA appears to stall Pol II at the promoter region and may regulate *IFNB1* and *CXCL8* expression by inhibiting transcription by Pol II in NPC. We hypothesize that EBV BART lncRNA expression modulates host gene expression and maintains EBV latency by interfering with histone methylation and acetylation processes. Aberrant expression of affected host genes mediated by BART lncRNA may lead to immune evasion, progression, and metastasis of NPC.

## Introduction

Epstein-Barr virus (EBV) infects more than 95% of the human population. Primary infection with EBV is usually asymptomatic, however, if infection first occurs in young adulthood, mononucleosis may develop (1). EBV remains latent in memory B cells and is kept in check by a competent immune surveillance system which continuously removes EBV-infected cells in which EBV has become activated from its stringent latency program (2). When the human immune system is compromised, latently EBV-infected cells may be transformed into lymphoblasts, which are characterized by increased cell proliferation, as seen in lymphoproliferative diseases. Reactivation of EBV occurs regularly *in vivo*, as demonstrated by virus shedding in the saliva of EBV-positive individuals. While latent infection of resting B cells with EBV and virus shedding from oral pharyngeal tissues normally cause no serious risk to the health of immunocompetent individuals, the presence of EBV can give rise to certain epithelial cancers, including nasopharyngeal carcinoma (NPC), when combined with genetic abnormalities or undefined environmental factors. We postulate that infection with activated EBV in nasopharyngeal epithelial cells predisposed toward establishment of viral latency may lead to the origin or development of NPC. The questions of how EBV-infected NPC cells can evade immune surveillance in apparently immune competent individuals and what the exact contribution of EBV is to the oncogenesis of NPC remain unanswered. However, in NPC cells EBV is known to turn off the expression of most of its latency genes which are critical for B cell transformation, presumably to avoid immune surveillance. But how does EBV maintain latency in NPC cells without these viral functions? It is likely that the mechanism regulating the EBV latency program in NPC cells also drives EBV oncogenesis.

In NPC cells, EBV mainly expresses EBNA1, EBV-encoded RNAs (EBERs), and elevated levels of *Bam*HI-A rightwards transcripts (BARTs). EBNA1 is essential for EBV replication and EBERs have been reported to play a role in antagonizing host antiviral innate immunity. While EBV LMP1 is a recognized viral oncogene, expression of LMP1 protein is not consistently observed in EBV-infected NPC cells. EBV BARTs were first identified as multi-spliced transcripts in NPC tissues and were later found to be expressed in all types of EBV-infected cells and EBV-associated tumors (3–6). However, no clear role could be attributed to BARTs in EBV infection until it was revealed that BARTs encode two clusters of EBV microRNAs with versatile functions in all forms of EBV latency (7). It is now clear that EBV BARTs comprise two groups of non-coding RNAs; a group of microRNAs (miRNAs) which are produced from introns prior to splicing and a complex family of alternatively spliced polyadenylated RNAs (7, 8). BART RNAs and miRNAs are both highly expressed in NPC, and to a lesser extent in EBV-positive gastric carcinoma and EBV-infected B cells. Our previous studies have characterized the promoters driving transcription of BARTs and show that this abundant transcription is driven by C/EBP and aberrant NF-κB signaling, and that BART miRNAs in turn modulate NF-κB activation through LMP1 in an auto-regulatory loop in NPC cells (9, 10). Expression of *C/EBP* is mainly found in epithelial cells while aberrant NF-κB signaling has been reported in NPC cells (10–12), which may contribute to the elevated level of BARTs in NPC cells. Alternative splicing of BARTs results in multiple spliced forms of BART RNA, with putative open reading frames in BARF0, RK-BARF0, RPMS1, and A73 (3, 13). However, attempts to identify proteins translated from these transcripts have been unsuccessful. Previous reports indicated that BART RNAs are restricted to the nucleus, which supports the idea that these BART RNAs may function as regulatory RNAs, rather than coding for a protein (14, 15). One recent study reported that ectopic expression of one isoform of BART RNA altered transcription of cellular genes in AGS cells, suggesting that BART RNAs may function as long non-coding RNAs (lncRNAs) (14). However, the role of BART RNAs in EBV latency and NPC, including whether they act as lncRNAs, is yet to be defined in detail.

To determine if EBV BART RNAs function as lncRNAs in NPC, this study first confirmed that BART RNAs are predominantly localized within the nucleus, as many lncRNAs tend to be nuclear. RNA-seq analysis revealed that knockdown of BART RNAs in NPC cells resulted in altered expression of genes associated with host immune/inflammatory responses, and oxidoreductase and cell adhesion activities, supporting the idea that they function as lncRNAs in NPC. Our data suggest that BART lncRNAs may affect host gene expression through epigenetic regulation and chromatin remodeling. Expression of the host transcription factor, Aiolos, is normally restricted to lymphoid cells, but it is aberrantly expressed in NPC and appears to be regulated by BART lncRNAs (16, 17). This study highlights a mechanism in which EBV expresses BART lncRNAs to modulate host gene expression, generating a cellular environment that supports EBV latency, and driving the oncogenic process in NPC.

## Materials and Methods

### Cell Lines and Cell Culture Conditions

Human embryonic kidney (HEK) 293T cells, CNE2, and HeLa-Bx1 cells were maintained in Dulbecco's Minimal Essential Medium (Gibco) supplemented with 10% fetal bovine serum (FBS) and 1% P/S. The EBV-positive NPC cell line C666-1 and the Burkitt's lymphoma lines Mutu III, Mutu I and DG-75 were grown in RPMI-1640 medium (Gibco) supplemented with 10% fetal bovine serum (FBS) and 1% P/S. The hTERT immortalized NP epithelial cell lines NP361-hTERT-EBV and NP460-hTERT-EBV were grown in a 1:1 mixture of Defined Keratinocyte-SFM (Gibco) and Epilife™ medium (Gibco) supplemented with 1% P/S. The EBV-positive and -negative gastric cancer cell line AGS-Bx1 and AGS, respectively, were cultured in F-12K Nutrient Mixture (Gibco) supplemented with 10% fetal bovine serum (FBS) and 1% P/S. Cells were cultured at 37°C with 5% CO_2_.

### Plasmids

The MAVS expression plasmid pEF-BOS MAVS was a gift from Kate Fitzgerald (Addgene, plasmid 27224). The pcDNA3-BART expression plasmid contains a full-length BART clone representing the major isoform of BART RNA, *RPMS1* (13). The BART clone, almost 4-kb in length, contains exons I, IIIa, IIIb, IV, V, VI, and VII, which was confirmed by sequencing. The oriPtL expression plasmid was created by amplifying and cloning the oriPtL sequence spanning nucleotides 7,143–9,247 of the EBV genome from cell line C666-1 (GenBank: KJ411974.1) into pcDNA3.

### ChIP Assay

Briefly, C666-1 cells transfected with LNA™ BART or negative control A GapmeRs (Exiqon) and HEK 293T cells transfected with pEF-BOS MAVS and pcDNA3-BART or empty vector were harvested after 48 h. The ChIP extract was sonicated into DNA fragments sized between 100- and 1000-bp using a Sonicator S-4000 (Misonix). For immunoprecipitation, 5 μg of rabbit anti-Pol II (sc-899, Santa Cruz Biotechnology) or 5 μg of normal rabbit IgG (sc-2027, Santa Cruz Biotechnology) was used and antibody-protein-DNA complexes were pulled-down using Dynabeads Protein A (Invitrogen). The level of immunoprecipitated DNA was determined by qPCR.

### Luciferase Reporter Assay

HEK 293T cells were seeded at a density of approximately 70% in 24-well plates a day before transfection, and subsequently co-transfected with 100 ng of pEF-BOS-MAVS, 500 ng of pcDNA3-BART or pcDNA3-oriPtL, and 100 ng of a Firefly luciferase reporter plasmid driven by the IFN-β promoter (Promega), using Lipofectamine® 2000 (Invitrogen). For data normalization purposes, 10 ng of the plasmid phRL-TK (Promega) expressing *Renilla* luciferase was co-transfected with the Firefly reporter plasmid in each experiment. Cells were harvested 2 days after transfection.

### Immunoblotting

Membranes were incubated overnight with primary antibodies in 3% milk in PBST. The antibodies used for immunoblotting included rabbit anti-IKZF3 (ab139408, Abcam), rabbit anti-CDK8 (A302-501A, Bethyl Laboratories), and mouse anti-β-tubulin (T8328, Sigma-Aldrich) at a 1:2,000 dilution and rabbit anti-SHC (ab24787, Abcam), rabbit anti-SEPT9 (sc-899, Santa Cruz Biotechnology), and mouse anti-MAVS (sc-166583, Santa Cruz Biotechnology) at a 1:1,000 dilution. Membranes were then incubated with IRDye700-labeled donkey anti-mouse or anti-rabbit or IRDye800-labeled donkey anti-mouse (LI-COR Biosciences) at a 1:5,000 dilution. Blots were detected using an Odyssey® Infrared Imaging System (LI-COR Biosciences).

### Reverse Transcription and qPCR Amplification

RNA was extracted from cells using RNAiso Plus reagent (TaKaRa) and reverse transcribed with random primers using the High-Capacity cDNA Reverse Transcription Kit (Applied Biosystems). Real-time PCR reactions were performed using SYBR Premix Ex Taq (Tli RNase H Plus) mix (Takara) in a LightCycler 480 instrument (Roche). Primers used in this study are listed in Supplementary Table 2 and gene expression was normalized to that of glyceraldehyde-3-phosphate dehydrogenase (GAPDH).

### RNA FISH and Immunofluorescence

Stellaris fluorescent *in situ* hybridization (FISH) probes were designed and purchased from Biosearch Technologies with Quasar 570 fluorophore to detect BART lncRNA and CAL Fluor Red 635 to detect GAPDH mRNA. FISH combined with indirect immunofluorescence (IF) was performed according to the Biosearch Technologies online protocol for sequential IF + FISH in adherent cells. Cells were fixed, permeabilized, and incubated overnight at 37°C with 125 nM FISH probes and a 1:100 dilution of primary antibody in hybridization buffer (100 mg/mL dextran sulfate, 10% formamide in 2X SSC). After incubation, cells were washed with wash buffer (10% formamide in 2X SSC) and then incubated with secondary antibodies conjugated with FITC at a 1:200 dilution in wash buffer for 30 min at 37°C. Finally, the cells were washed and mounted using mounting medium with DAPI (Vectashield) and the signals were visualized using a Carl Zeiss LSM 710 confocal microscope.

### Histone Acetyltransferase Activity Assay

Nuclear extracts were prepared by lysing cells in phosphate buffered saline (PBS) containing 1% NP-40 and after a short centrifugation the supernatant (cytoplasmic fraction) was removed. The nuclear extracts were washed twice with 1% NP-40 in PBS and lysed using RIPA lysis buffer. HAT assay was performed using a Histone acetyltransferase activity assay kit (ab65352, Abcam) according to the manufacturer's protocol. For each assay 30 μg nuclear extract was used and the OD was measured at 450 nm at 1-h intervals.

### RNA Sequencing

Two total RNA extracts from C666-1 cells were independently prepared 2 days after transfection with BART or control GapmeRs, using the NucleoSpin RNA II kit (Macherey Nagel) in accordance with the manufacturer's instructions. Four micrograms of total RNA was used as starting material for ribosomal RNA (rRNA) depletion, performed using the human Ribo-Zero Gold rRNA Removal Kit (Illumina). Library preparation and sequencing was performed by the University of Hong Kong Center of Genomic Sciences using the HiSeq SBS Kit v4 (Illumina) and the HiSeq PE Cluster Kit v4 cBot (Illumina) on an Illumina HiSeq 1500 instrument for pair-end 101 bp sequencing. Sequencing reads were first filtered for adapter and low-quality sequences, followed by retaining only reads with a read length ≥ 40 bp by using Cutadapt (http://code.google.com/p/cutadapt/). Low quality reads were defined as reads with more than 5% unknown bases (N) and reads having more than 50% of bases with a quality value ≤ 10. Sequence reads were subsequently filtered for rRNA sequences and the remaining reads were used for downstream analysis.

### Computational and Statistical Analyses

Filtered RNA sequencing reads were first aligned to the human genome (GRCh38, gencode v24), downloaded from GENCODE (18), and the remaining unaligned reads then aligned to the wild type EBV genome (GenBank: NC_007605) using STAR version 2.5.2a (19). Quantification of expression and identification of differential gene expression was performed using Partek Genomics Suite (version 6.6). Annotation for the EBV genome was manually edited into the GenBank record before importing alignment files into Partek Genomics Suite. Statistical analysis of RT-qPCR data was performed using GraphPad Prism 5 software. Significance values (*P*-values) were calculated using the two-tailed Student's *t*-test, with Welch's correction, where appropriate.

## Results

### BART lncRNA Is Localized in the Nucleus of EBV-Infected Cells

**Figure 1:**
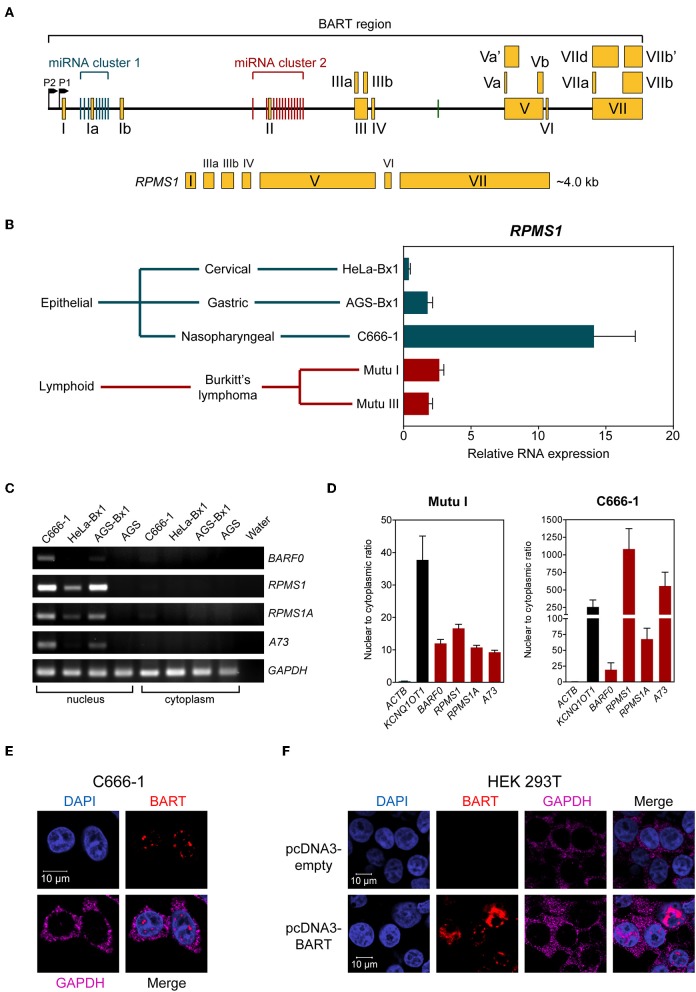
BART RNA expression and localization. **(A)** Position and structure of the BART RNA *RPMS1* variant. Exons are indicated by yellow boxes while the black line in between represents intronic sequences, with miRNA clusters indicated by green (cluster 1) and red (cluster 2) vertical bars. **(B)** Relative expression of *RPMS1* RNA in various EBV-positive cell lines with different latency programs, analyzed by RT-qPCR. Gene expression is shown as RNA expression relative to that of *GAPDH*. The average and SEM of three independent experiments are shown. **(C)** RT-PCR and agarose gel analysis of nuclear and cytoplasmic RNA from different EBV-positive epithelial cell lines. AGS is an EBV-negative cell line, included as a control. **(D)** Nuclear to cytoplasmic ratio of BART RNA variants in Mutu I and C666-1 cell lines, analyzed by RT-qPCR. *KCNQ1OT1* was analyzed as a nuclear transcript control, and *ACTB* was analyzed as a cytoplasmic control. Data shown represent the average and SEM of three independent experiments. **(E,F)** RNA FISH showing localization of BART RNA in C666-1 and HEK 293T cell lines, respectively. RNA FISH probes against *GAPDH* mRNA were used as a cytoplasmic control.

The EBV *Bam*HI-A region encodes BART-miRNAs and BART RNA, which are derived from the introns and exons, respectively (Figure 1A). EBV BART RNA comprises multiple spliced forms of transcripts; *RPMS1* BART RNA has a size of about 4 kb and is the most abundant of the BART transcript species expressed in NPC cells (3). We and others have previously described EBV-derived miRNAs in EBV positive epithelial and B cell lines (9, 20). Here we compared the levels of the BART RNA, *RPMS1*, in the C666-1 NPC cell line and other EBV-infected cell lines. EBV expresses the highest levels of BART *RPMS1* in C666-1, which is naturally infected with EBV, while relatively lower levels of *RPMS1* are detected in HeLa-Bx1 and AGS-Bx1, which have been artificially infected with EBV *in vitro*, and in both latency I and III Mutu cell lines, which are derived from Burkitt's lymphoma (Figure 1B). It has been suggested that BART RNAs may represent a group of viral lncRNAs with as yet undefined functions (14). BART lncRNAs are predominantly detected in the nuclear fractions of EBV-harboring C666-1 and EBV-infected AGS cell lines (15). We confirmed the localization of BART lncRNAs in the nuclear fraction of all tested EBV-positive cell lines by quantitative RT-PCR (Figures 1C,D). The nuclear localization of BART lncRNA was further analyzed by RNA fluorescent *in-situ* hybridization (FISH) assay in C666-1 cells and pcDNA3-BART-transfected HEK 293T cells. RNA FISH clearly demonstrated distinctive dotted patterns of BART RNA exclusively in the nucleus (Figures 1E,F). These results further confirm that BART lncRNA is expressed at elevated levels in the nucleus of NPC cells.

### GapmeR-Mediated Targeting of BART lncRNA in C666-1 Cells

**Figure 2:**
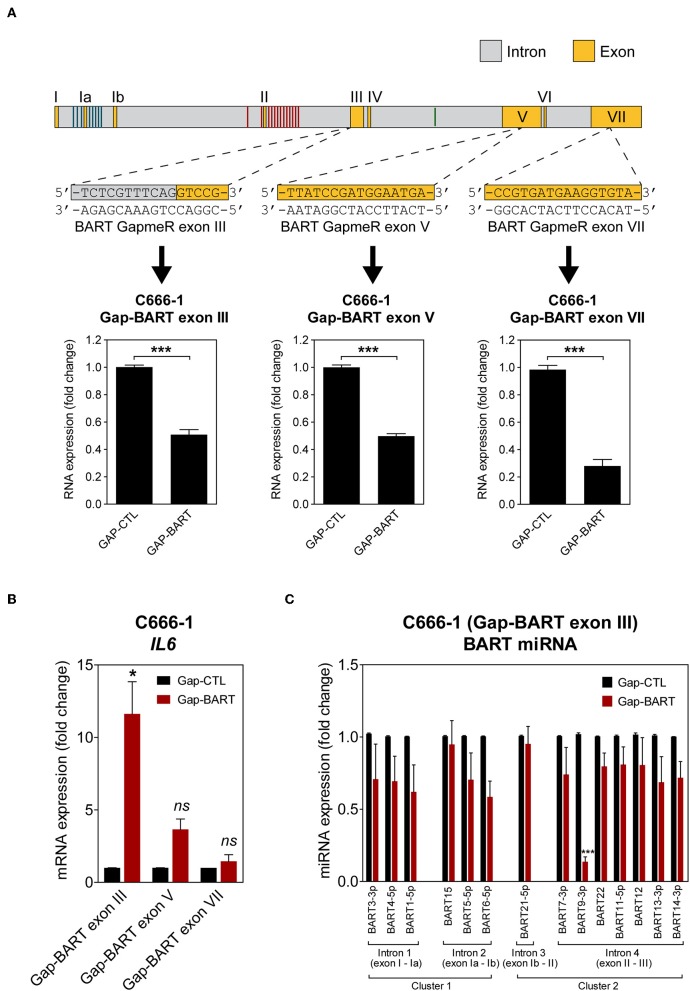
Targeting of BART RNA with LNA™ longRNA GapmeRs. C666-1 cells were transfected with 100 nM LNA™ longRNA GapmeRs and RNA extracted 48 h later. **(A)** Three GapmeRs targeting different sequences within the BART region were analyzed to check their ability to cleave their target sequence and reduce BART RNA expression. Gene expression is shown as fold change in RNA expression relative to that of *GAPDH*. **(B)** Validation of the effect of GapmeRs targeting BART RNA on host gene expression in C666-1 cells. Expression of *IL6* was estimated by RT-qPCR following Gap-BART targeting of exons III, V, and VII. Gene expression is shown as fold change in RNA expression relative to that of *GAPDH*. **(C)** Analysis of BART miRNA expression in C666-1 cells after Gap-BART transfection. Expression is shown as fold change in miRNA expression relative to that of miR-Hsa-16. Results represent the average and SEM of three independent experiments. Statistical significance was calculated using the two-tailed Student's *t*-test. ^*^P < 0.05, ^***^P < 0.001, ns, not significant.

To characterize the effect of BART RNA on host gene expression in NPC cells, knockdown of BART RNA was achieved by targeting it for GapmeR-mediated RNase H cleavage to disrupt its function. Three different GapmeRs targeting different locations within the BART region were tested in C666-1 cells, and all showed equal cleavage efficiency (Figure 2A). Pilot experiments with the *RPMS1* expression plasmid revealed that BART lncRNA overexpression and *IL6* gene expression were negatively correlated. Expression of *IL6* mRNA was used to determine the impact of targeting BART RNA with GapmeRs in C666-1. Consistently, GapmeR targeting of the splicing junction at the start of exon III, exon V, and VII all caused the significant change in host *IL6* gene expression. Gap-BART exon III was then used in subsequent experiments to knockdown endogenous expression of BART RNA in C666-1 cells (Figure 2B). The expression of various BART miRNAs from BART miRNA clusters 1 and 2 was also analyzed to determine how targeting BART RNA affects their expression. Of the 21 BART miRNAs tested, the 14 miRNAs with the greatest changes in expression are shown in Figure 2C. Only miR-BART6-5p and miR-BART9-3p showed clearly lowered expression, with only the miR-BART9-3p result being statistically significant; other tested BART miRNAs demonstrated a moderate and statistically insignificant reduction in expression following knockdown of BART lncRNA (Figure 2C). Since the BART GapmeR used does not target a region near the sites from which BART miRNAs are produced, it is likely that miRNA production remains largely intact.

### BART lncRNA Modulates Host Gene Expression

Using the GapmeR knockdown strategy described above, we analyzed the transcriptional profile in C666-1 cells treated with either Gap-BART exon III (from here on referred to as Gap-BART) or control GapmeRs (Gap-CTL) by RNA-seq. Sequence reads generated by pair-end 101-bp sequencing on the Illumina HiSeq 1500 sequencer were first aligned to the human genome (hg38), after which the unmapped reads were manually aligned to the wild type EBV genome (GenBank: NC_007605). The vast majority of filtered reads, between 94.9 and 95.5%, were mapped to the human genome, while only 0.11–0.30% were mapped to EBV (Supplementary Tables 1A,B). The low frequency of EBV sequences is consistent with the fact that EBV infection in NPC C666-1 is latent. Notably, transcripts mapped to EBV were mainly derived from the *Bam*HI-A region (Supplementary Figure 1).

**Figure 3:**
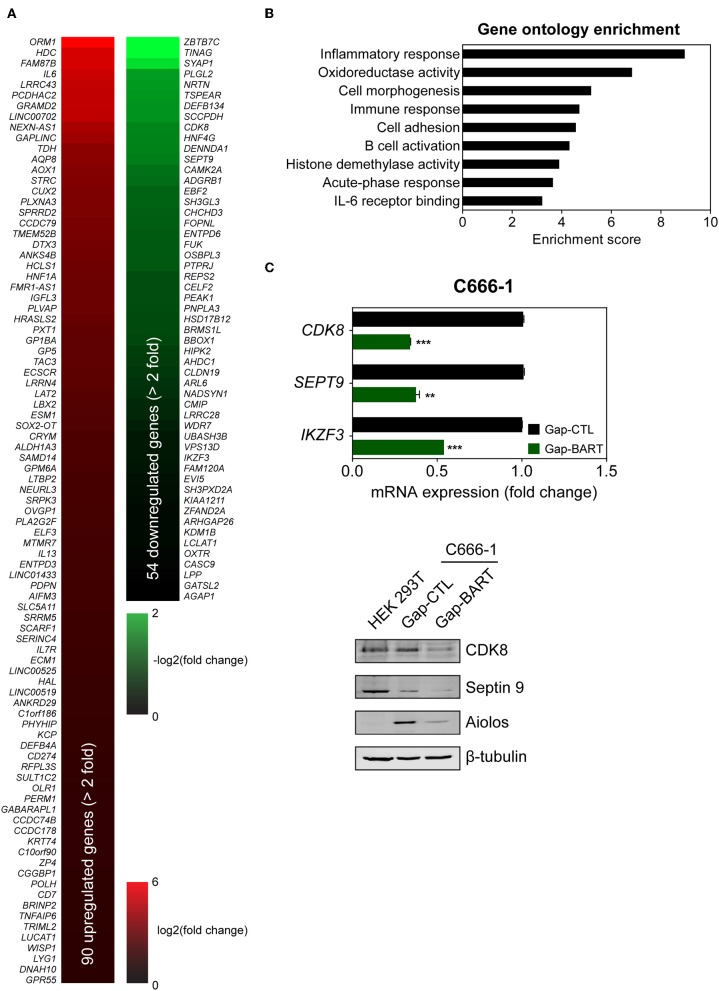
Differential host gene expression following BART lncRNA knockdown, analyzed by RNA-seq. C666-1 cells were transfected with 100 nM LNA™ longRNA GapmeRs and RNA extracted for RNA-seq analysis 48 h later. **(A)** Upregulated (red) and downregulated (green) genes with a more than 2-fold change in expression with an FDR threshold of 0.1. **(B)** Gene ontology (GO) enrichment analysis of the up- and down-regulated genes was performed to group genes affected by BART lncRNA-targeting according to their involvement in biological processes and molecular functions. **(C)** RT-qPCR and immunoblot validation of mRNA and protein expression of *CDK8, SEPT9*, and *IKZF3*, which are downregulated following knockdown of BART lncRNA. Gene expression is shown as fold change in mRNA expression relative to that of *GAPDH*. C_P_ value > 35 were considered unreliable. The average and SEM of three independent experiments are shown. ^**^P < 0.01, ^***^P < 0.001.

Targeting BART lncRNA with GapmeRs resulted in the downregulation of 54 genes and the upregulation of 90 genes in C666-1 cells (>2-fold change for both upregulation and downregulation), when a false discovery rate (FDR) threshold of <0.1 was applied (Figure 3A). The influence of BART RNA on expression of other genes, together with its nuclear location, indicates that it most likely functions as lncRNA in NPC, and will henceforth be referred to as BART lncRNA. Differentially expressed genes identified through RNA-seq analysis were further analyzed using Partek Genomics Suite (version 6.6), which revealed gene ontology groupings with high enrichment scores and low *P*-values that are relevant to cancer (e.g., cell adhesion) and EBV infection (e.g., inflammatory and immune responses) (Figure 3B). The largest functional group consists of genes involved in the inflammatory response, which were all upregulated when BART lncRNAs were knocked down using GapmeRs. Similarly, most of the genes related to the immune response (6/7) and cell adhesion (6/8) were upregulated following targeting of BART lncRNA. Representative genes from the four gene ontology groups most relevant to EBV infection and cancer, namely inflammatory response, oxidoreductase activity, immune response, and cell adhesion were examined by quantitative RT-PCR (RT-qPCR) analysis to validate their response to BART lncRNA-knockdown (Supplementary Figure 2).

Among the differentially expressed genes, three genes from the downregulated group (*CDK8, SEPT9*, and *IKZF3*) were validated by both RT-qPCR and immunoblot (Figure 3C) to confirm the effect of BART lncRNA on the expression of these genes. These affected genes are associated with tumor progression, migration, and aberrant signaling in cancers (21–25). In particular, *IKZF3*, which encodes Aiolos and is normally not expressed in epithelial cells (26), was identified as being a potentially important target of BART lncRNA and was further examined in subsequent experiments.

### Aiolos Is Expressed in NPC and Regulated by BART lncRNA

**Figure 4:**
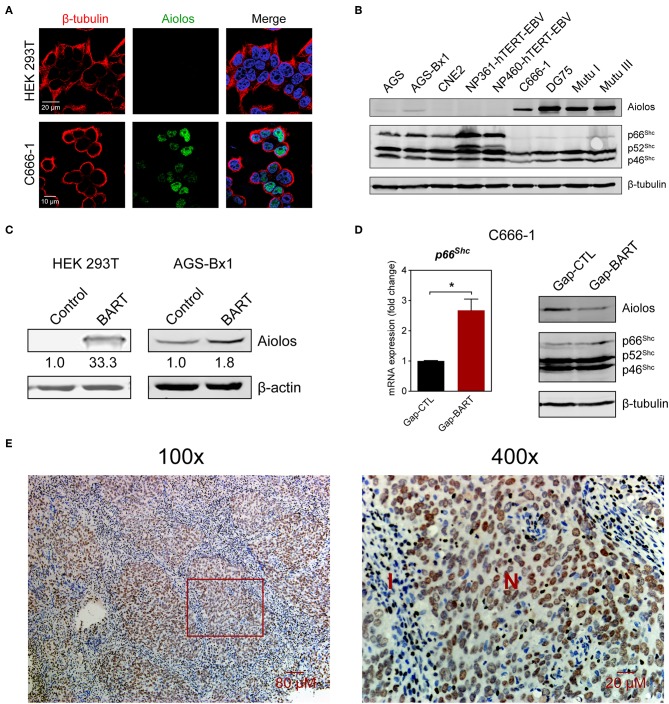
Aiolos expression in NPC. **(A)** Aiolos protein expression was analyzed by indirect immunofluorescence in HEK 293T and C666-1 cells. **(B)** Immunoblot analysis showing Aiolos and p66^Shc^ expression in various cell lines. AGS, AGS-Bx1, CNE2, NP361-hTERT-EBV, NP460-hTERT-EBV, and C666-1 are epithelial cell lines, while DG75, Mutu I, and Mutu III are Burkitt's lymphoma cell lines. All cell lines are EBV-positive, except for AGS, CNE2, and DG75. **(C)** Effect of BART lncRNA on Aiolos expression. A plasmid containing one of the major species of BART lncRNAs (*RPMS1*) was transfected into HEK 293T and AGS-Bx1 cells. **(D)** Effect of targeting BART lncRNA with GapmeRs on levels of p66^Shc^ mRNA expression and Aiolos protein expression. The average and SEM of three independent experiments are shown. Gene expression is shown as fold change in mRNA expression relative to that of *GAPDH*. ^*^P < 0.05. **(E)** Immunohistochemistry for Aiolos was performed on 26 NPC biopsies. A representative image shows Aiolos staining (brown) in NPC cells at 100× and 400× magnification. I, cellular infiltrate; N, NPC cells.

Aiolos, encoded by the *IKZF3* gene, is a lymphocyte-restricted transcription factor which is normally not expressed in epithelial cells (26). However, Aiolos expression has been detected in various malignant solid tumor cell lines and was found to downregulate adhesion-related genes in lung cancer cells (17). Since BART lncRNA expression seems to positively regulate *IKZF3* expression in C666-1 cells and negatively regulate cell adhesion-associated genes like *PCDHAC2* (Figures 3A,C), we first determined whether Aiolos is truly expressed in NPC cells. Indirect immunofluorescence with HEK 293T and C666-1 cells confirmed that Aiolos is expressed in the nucleus of NPC cells, but not in HEK 293T cells (Figure 4A). This result was further supported by immunoblotting, where moderate Aiolos expression could only be detected in C666-1 cells, with barely perceptible expression in AGS-Bx1 and none in any of the other epithelial cell lines tested, which included both EBV-positive and -negative cell lines (Figure 4B). As expected, both EBV-positive and -negative Burkitt's lymphoma cell lines exhibited high levels of Aiolos protein. In addition, all Aiolos-expressing cell lines exhibited no or very low *p66*^Shc^ expression levels, which is consistent with a previous observation in lung cancer cells (17), and suggests that expression of Aiolos may also facilitate anchorage independence in NPC. This inverse relationship between *IKZF3* and *p66*^*Shc*^ expression was also observed at the mRNA level (Supplementary Figures 3A,B). To further confirm regulation of Aiolos expression by BART lncRNA, we examined HEK 293T cells and AGS-Bx1 cells after transfection with a BART lncRNA expression vector. Compared to C666-1, AGS-Bx1 normally expresses relatively low levels of BART lncRNA and Aiolos (Figures 1B, 4B). When transfected with pcDNA3-BART, expression of Aiolos is activated in HEK 293T cells, and significantly enhanced in AGS-Bx1 cells (Figure 4C). While GapmeR targeting of BART lncRNA not only results in downregulation of *IKZF3* expression, but also upregulates *p66*^*Shc*^ mRNA expression, increased expression of p66^Shc^ at the protein level is not apparent (Figures 4A,D). Next, we examined Aiolos expression by immunohistochemistry and observed positive staining in about 60% of NPC biopsies (17/26). Aiolos staining was apparent in tumor cells, but not in infiltrated non-cancerous cells (Figure 4E). It seems that EBV BART lncRNA may be involved in the regulation of host gene expression associated with anchorage independence in NPC cancers. These results suggest that modulation of Aiolos expression and associated pathways by EBV BART lncRNA may play an important role in NPC oncogenesis.

### BART lncRNA Modulates Immune-Related Genes

**Figure 5:**
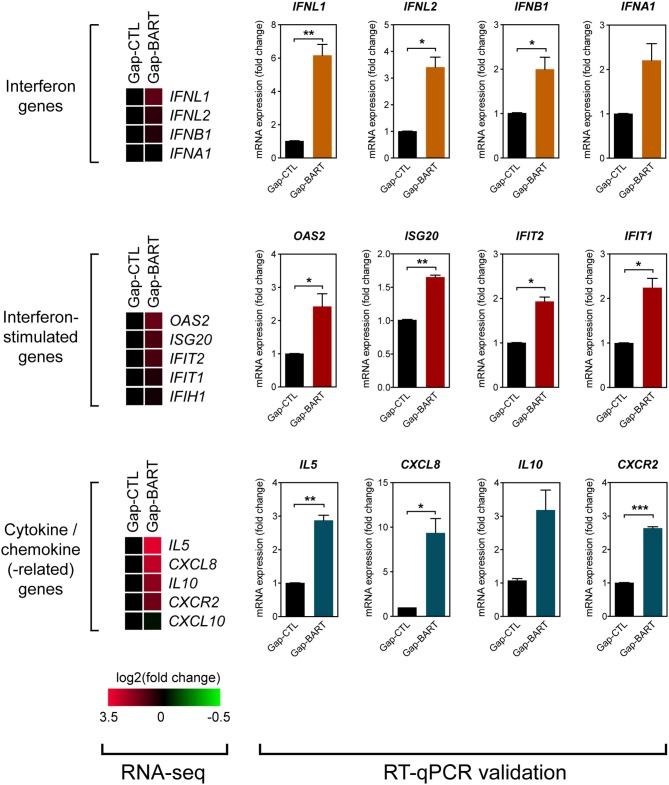
Targeting BART lncRNA affects immune-related gene expression. Differential expression of various interferon, interferon-stimulated, cytokine and chemokine genes not fitting the RNA-seq analysis criteria (>2-fold up- or down-regulation, 0.1 FDR) was analyzed by RT-qPCR. Gene expression is shown as fold change in mRNA expression relative to that of *GAPDH*. Results represent the average and SEM of three independent experiments. ^*^P < 0.05, ^**^P < 0.01, ^***^P < 0.001.

Besides modulating expression of *IKZF3* and a variety of cell adhesion genes, we also found that BART lncRNA affects inflammatory and immune response-related genes, such as *IL6, IL13*, and *IL7R* (Figure 3A). Analysis of the RNA-seq data revealed that several immune-related genes were differentially expressed following targeted knockdown of BART lncRNA, and although the differences were not statistically significant in the RNA-seq analysis, they could be validated by RT-qPCR (Figure 5). Expression of two type III (*IFNL1* and *IFNL2*) and two type I (*IFNB1* and *IFNA1*) interferon genes was clearly upregulated, and various interferon-stimulated genes (ISGs) (*OAS2, ISG20, IFIT2*, and *IFIT1*), cytokine genes (*IL5, CXCL8*, and *IL10*), and a chemokine-related gene (*CXCR2*) were also upregulated by BART lncRNA-specific GapmeR treatment in C666-1 cells, indicating that EBV BART lncRNA may modulate the host immune response to facilitate immune evasion. The *CXCL8* gene, which encodes IL-8, and its corresponding receptor gene, *CXCR2*, are interesting targets of BART lncRNA because IL-8 is regulated by LMP1, functions as a chemotactic factor, and has been reported to promote metastasis of NPC by inducing epithelial-mesenchymal transition and downregulating E-cadherin through activation of Akt signaling (27, 28). While knockdown of BART lncRNA seems to cause upregulation of most interferons, ISGs, cytokines, and chemokine-related genes, it is interesting to note that *CXCL10* and *IFIH1*, which encodes MDA5, a cytoplasmic sensor of viral nucleic acids in host antiviral responses, were downregulated.

**Figure 6:**
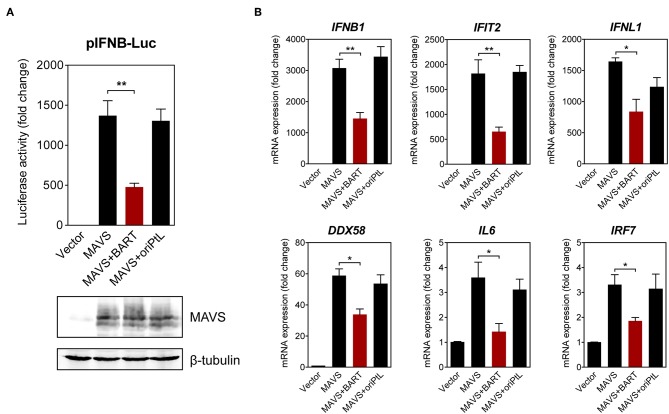
BART lncRNA expression modulates immune-related gene expression. **(A)** HEK 293T cells were co-transfected with an empty vector or an MAVS expression vector, with or without a BART or oriPtL expression vector, plus an IFN-β promoter reporter. Luciferase activities are expressed as fold change in luciferase activity, calculated by normalizing firefly/renilla ratios to the vector control. The expression of MAVS and β-tubulin was detected by immunoblotting using specific antibodies. **(B)** RT-qPCR analysis of the expression of various immune-related genes following transfection of HEK 293T cells with an empty vector or an MAVS expression vector, with or without a BART or oriPtL expression vector. Gene expression is shown as fold change in mRNA expression relative to that of *GAPDH*. The average and SEM of three independent experiments are shown. ^*^P < 0.05, ^**^P < 0.01.

We then sought to confirm the effect of BART lncRNA on the expression of immune-related genes by overexpressing the BART lncRNA isoform *RPMS1* together with mitochondrial anti-viral signaling protein (MAVS) in HEK 293T cells. A plasmid expressing the EBV-encoded nuclear lncRNA, *oriPtL*, was used as a control in these experiments (29). We found that BART lncRNA significantly inhibited MAVS-induced IFN-β promoter activity, while *oriPtL* did not show any effect (Figure 6A). Analysis of mRNA levels by RT-qPCR showed that expression of BART lncRNA downregulates a range of MAVS-induced interferons and ISGs in HEK 293T cells (Figure 6B). These results confirm an immunomodulatory role for BART lncRNA, consistent with the data from C666-1 cells treated with GapmeRs targeting EBV BART lncRNA.

### BART lncRNA Inhibits Gene Expression by Affecting Transcription by RNA Polymerase II

**Figure 7:**
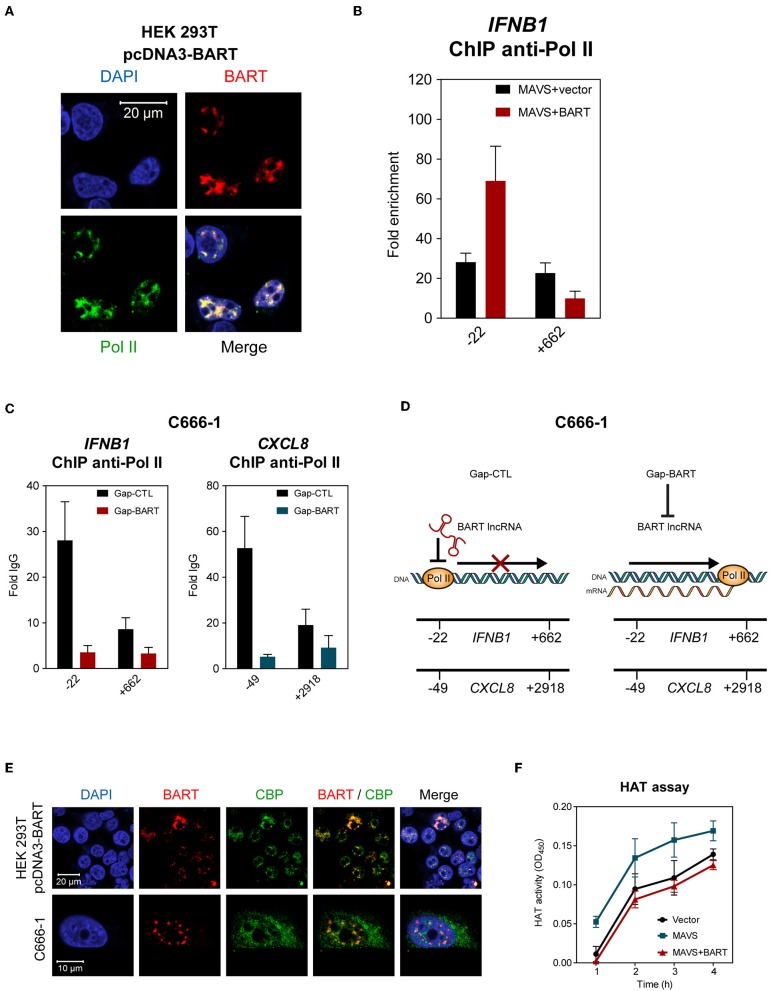
BART lncRNA affects gene transcription through Pol II. **(A)** Combined RNA FISH and indirect immunofluorescence was performed to detect BART lncRNA and Pol II expression in HEK 293T cells transfected with the BART expression vector. **(B)** ChIP analysis of *IFNB1* promoter (−22) and downstream (+662) DNA with Pol II antibodies in HEK 293T cells where MAVS was expressed alone, or together with BART lncRNA. The average and SEM of three independent experiments are shown. **(C)** ChIP analysis of *IFNB1* and *CXCL8* promoter and downstream DNA with Pol II antibodies in BART lncRNA-knockdown C666-1 cells. The average and SEM of three independent experiments are shown. **(D)** A model suggesting how *IFNB1* and *CXCL8* expression is increased following knockdown of BART lncRNA. **(E)** Combined RNA FISH and indirect immunofluorescence analysis of HEK 293T cells transfected with the BART expression vector and C666-1 cells, showing expression of BART lncRNA and CBP. **(F)** MAVS-induced HAT activity in HEK 293T cells transfected with a MAVS expression vector, with or without a BART expression vector. For transfection experiments, extracts were obtained 48 h after transfection. The average and SEM of three independent experiments are shown.

The results described above indicate that BART lncRNA regulates expression of host genes associated with innate immunity and the oncogenesis process, so we then investigated how BART lncRNA regulates host gene expression. Using indirect immunofluorescence and RNA FISH, we found that BART lncRNA co-localizes with RNA polymerase II (Pol II) in HEK 293T cells (Figure 7A), indicating that BART lncRNA may play a role in transcriptional regulation by interacting with the Pol II complex. To verify that BART lncRNA may be involved with the Pol II-promoter complex, HEK 293T cells were transfected with pcDNA3-BART, followed by chromatin-immunoprecipitation (ChIP) of the promoter or coding regions of the *IFNB1* gene, which is negatively regulated by BART lncRNA. ChIP analysis with anti-Pol II showed a higher enrichment at the promoter region (−22) of the *IFNB1* gene, but this higher enrichment was not observed downstream near the end of the *IFNB1* gene (+662) (Figure 7B). It is tempting to hypothesize that elongation of transcription by Pol II from the promoter region (−22) may be blocked or stalled, resulting in reduced transcription of the BART lncRNA-targeted gene. To further test this hypothesis, we analyzed Pol II occupation at the promoter regions of *IFNB1* and *CXCL8* in C666-1 cells and observed that knockdown of BART lncRNA resulted in significantly less Pol II enrichment at the promoters of both genes (Figure 7C). The reduced enrichment of Pol II following targeting of BART lncRNA with GapmeRs is in line with the increase in *IFNB1* and *CXCL8* gene expression shown in Figure 5. These findings suggest a mechanism by which BART lncRNA can downregulate gene expression by interfering with Pol II-mediated transcription (Figure 7D).

To further examine our hypothesis that EBV BART lncRNA is involved in epigenetic regulation of gene expression, we explored a possible association between BART lncRNA and the CREB-binding protein (CBP), which interacts with various transcription factors. CBP belongs to the p300/CBP co-activator family and plays a key role in the transcriptional activation of *IFNB1* and many other cellular genes. In HEK 293T cells transfected with pcDNA3-BART, RNA-FISH, and co-staining of CBP showed that BART lncRNA and CBP co-localize in the nucleus (Figure 7E). Similarly, co-localization of BART lncRNA and CBP was observed in C666-1 cells which harbor EBV and express abundant levels of BART lncRNA endogenously (Figure 7E). These results suggest a mechanism by which BART lncRNA regulates host gene expression through interaction with CBP in EBV infected cells. To further test this contention a histone acetyltransferase (HAT) assay was performed, which showed that BART lncRNA expression can inhibit MAVS-induced HAT activity, further indicating that BART lncRNA may play a role in regulating chromatin remodeling during the gene transcription process (Figure 7F).

## Discussion

EBV is recognized as one of the etiological factors for nasopharyngeal carcinoma, in addition to genetic predisposition and putative environmental factors (30). While considerable attention has been focused on oncogenesis in EBV-associated lymphomas, the role of EBV in nasopharyngeal carcinoma is less well-understood. Studies into the role of EBV in NPC may have been hampered by the perception that NPC is a rare cancer, a lack of suitable *in vitro* and *in vivo* models and, most importantly, the fact that EBV expresses very few viral proteins in NPC cells, despite all cancer cells harboring the EBV genome. Nevertheless, it has long been known that EBV expresses elevated levels of BART non-coding RNAs, including miRNAs, and lncRNAs, in NPC cells (3, 31). The biological significance of EBV BART miRNA has been extensively analyzed and various functions associated with cell growth, immune evasion and anti-apoptotic activities have been revealed or are being actively explored (32). However, little is known regarding the role of BART lncRNAs expressed from the same transcripts. Here we present results from analysis of RNA expression profiles from NPC C666-1 cells where BART lncRNA is knocked down. Our findings show that BART lncRNA is involved in modulation of host cell expression of genes involved in cell adhesion, and those encoding interferons, ISGs, cytokines, and chemokines. Notably, BART lncRNA was found to activate expression of *IKZF3* (Aiolos), which is normally restricted to lymphocytes. We further showed that nuclear BART lncRNA associates with Pol II and the CBP/p300 complex. These results suggest that EBV BART lncRNA may play a role in epigenetic modulation of host gene expression through interaction with the host DNA methylation machinery and chromatin remodeling process during gene transcription.

EBV adopts different latency programs to evade immune surveillance *in vivo*. The hallmark of EBV latency in NPC is expression of elevated levels of EBV BARTs, including miRNAs, and lncRNAs. It is possible that EBV BARTs perform latency-associated functions in NPC cells that are performed by EBV latency proteins in B cells. Advances in recognizing the function of lncRNAs in modulating gene expression has opened up new directions for exploring the biological significance of EBV BARTs in EBV infection and EBV-associated tumors. The expression of one of the isoforms of BART lncRNA can modulate cellular gene expression in a manner similar to that of EBV infection in AGS cells (33). Cellular lncRNAs have been found to interact with chromatin, protein, and RNA in both the nucleus and cytoplasm to modulate gene expression in cancer pathways (34). We have confirmed nuclear localization of BART lncRNA in both epithelial and lymphoid EBV-infected cell lines in different forms of latency by cellular fraction and RNA FISH analyses, supporting the idea that BART RNAs function as non-protein-coding transcripts in the nucleus. It seems likely that EBV has developed a strategy to shut off expression of most viral proteins to avoid immune surveillance, while utilizing BART lncRNAs to modulate host gene expression and enable establishment of latency in NPC cells. Consequently, this altered cellular gene expression may generate an environment that drives the oncogenesis process in NPC.

One of the interesting findings in this study is that *IKZF3* expression is downregulated upon knock down of BART lncRNA. Aiolos, encoded by *IKZF3*, is a member of the *Ikaros* zinc-finger protein family and its expression is usually restricted to lymphoid cells (26). Expression of Aiolos is accompanied by a sustained loss of lymphocyte adhesion to its matrix-rich microenvironment, enabling lymphocyte entry into the bloodstream and subsequent circulation to other organs (35). High levels of Aiolos expression have been reported in both liquid and solid tumors, where it promotes tumor cell survival and acts as an epigenetic driver of lymphocyte mimicry in metastatic epithelial cancers (24, 36). Aiolos promotes anchorage independence in lung cancer by downregulating several adhesion-related genes, and also by blocking anoikis through the silencing of the anchorage reporter gene *p66*^*Shc*^, an isoform of the *SHC1* gene (17). Notably, we detected Aiolos expression *in vitro* in C666-1 cells and *in vivo* in NPC biopsies, but not in many other epithelial cell lines (Figure 4B). We suspected that expression of Aiolos may be caused by BART lncRNA, and indeed found that ectopic expression of BART lncRNA induces Aiolos expression in HEK 293T cells and upregulates expression of Aiolos in AGS-Bx1 cells. Apart from BART lncRNA expression, high NF-κB and STAT3 activity also occurs in NPC cells (11, 37, 38); these two cell signaling pathways are known to activate the *IKZF3* promoter and may also contribute to Aiolos expression (16, 39). The upregulation of adhesion genes, as well as *p66*^*Shc*^, following targeting of BART lncRNA may therefore be explained in part by lower expression of Aiolos (Figure 4D). It seems possible that expression of BART lncRNAs inhibits cell adhesion and anoikis and may thereby facilitate anchorage independence and metastasis of cancer cells. Still unanswered is how altered cellular expression of genes, such as *IKZF3*, may contribute to the EBV latency program in NPC cells.

Most data obtained in this study seems to suggest that EBV BART lncRNA has a role in epigenetic activation. Septin 9, a well-established biomarker for colorectal cancer that is hyper-methylated in several cancers (21), is upregulated by EBV BART lncRNA. However, epigenetic suppression of *GAPLINC, DTX3*, and *ELF3* were also observed (Figure 3A). The biological significance of the epigenetic modulation of these cancer-associated genes needs to be further evaluated. The role of BART lncRNA in epigenetic regulation of host genes is further supported by evidence that BART lncRNA associates with p300/CBP and Pol II in the nucleus. Both *IFNB1* and *CXCL8* genes were upregulated in C666-1 cells by knocking down BART lncRNA; this also resulted in increased Pol II occupation at the promoter region, but not near the 3' end of these genes. It seems that BART lncRNA may stall Pol II at the promoter region, preventing transcription of the targeted genes, and resulting in lower gene expression. Other mechanisms of BART lncRNA regulation may involve histone methylation and acetylation through interaction with p300/CBP. Further studies are necessary to reveal the molecular basis of epigenetic regulation of host genes by BART lncRNA, and how consequently altered cellular gene expression may facilitate EBV latency and drive oncogenesis in EBV-associated tumors.

Our findings clearly suggest that BART lncRNAs are involved in epigenetic regulation of host gene expression in NPC. While more work needs to be done to fully characterize the role of BART lncRNA in EBV-associated tumors, it seems reasonable to postulate that EBV adopts a strategy to turn off expression of most antigenic latency proteins and instead express abundant levels of BART lncRNA to suppress the immune response in NPC cells. In NPC cells, most of the latent genes are highly methylated with only EBNA1 being expressed. BART lncRNA functions as an epigenetic modulator to generate a microenvironment that is conducive to EBV latency in NPC. Consequently, aberrant expression of genes mediated by BART lncRNA may lead to evasion of the immune response, cancer progression, and metastasis in NPC.

## Data Availability Statement

RNA-seq data has been uploaded to NCBI: https://www.ncbi.nlm.nih.gov/sra/PRJNA556573. This data can be accessed using the accession number: PRJNA556573.

## Author Contributions

RV and HC conceived the study and designed the experiments. RV, ST, BM, JL, SH, JZ, and YC performed experiments. RV, S-WT, ML, and HC analyzed data. RV and HC wrote the paper.

### Conflict of Interest

The authors declare that the research was conducted in the absence of any commercial or financial relationships that could be construed as a potential conflict of interest.
