# Supplementary material for: Epstein-Barr Virus BART Long Non-coding RNAs Function as Epigenetic Modulators in Nasopharyngeal Carcinoma
Source: Front Oncol. 2019 Oct 22;9:1120. doi: 10.3389/fonc.2019.01120 (PMC6817499; doi:10.3389/fonc.2019.01120)
Supplement: Supplementary file 1 [file Data_Sheet_1.pdf]

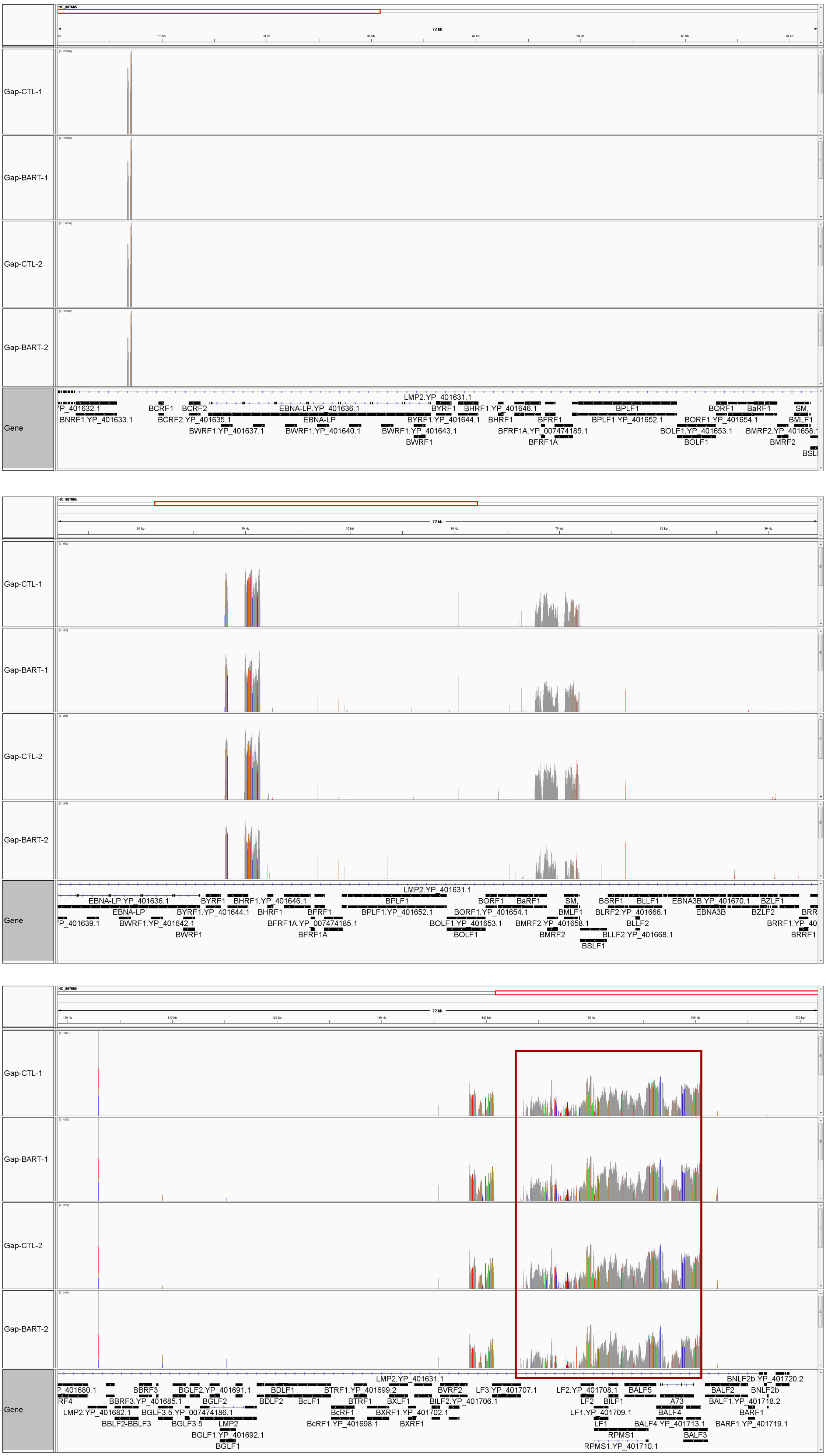

Supplementary figure 1 RNA-seq reads aligned to the wild type EBV genome confirm extensive transcription from the *Bam*HI-A region (highlighted by red square).

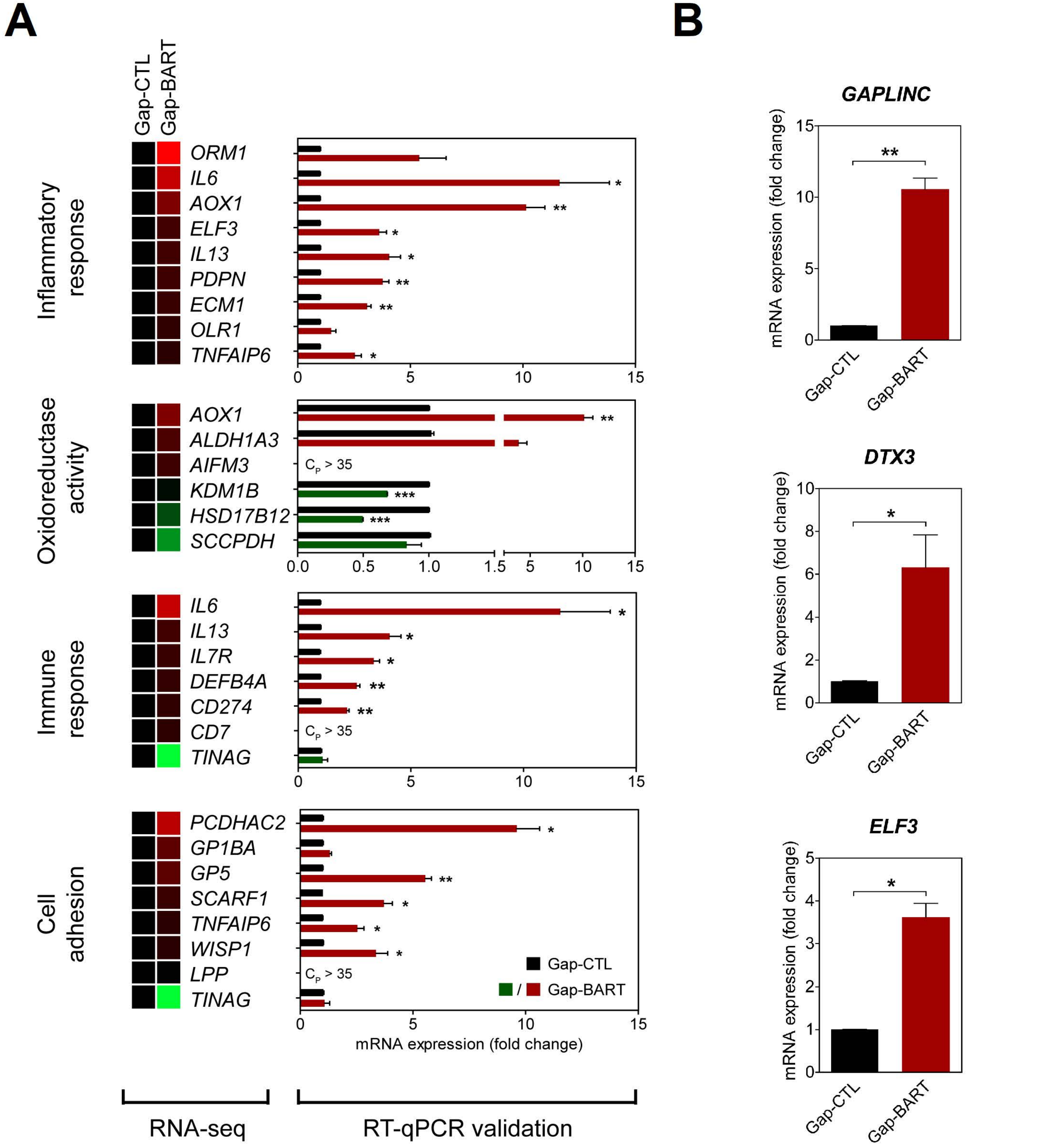

Supplementary figure 2 (A) RT-qPCR validation of differentially expressed genes from four groups identified by GO enrichment analysis which may play a role in EBV infection and NPC tumor progression. (B) RT-qPCR validation of *GAPLINC*, *DTX3* and *ELF3*, which are upregulated by knockdown of BART lncRNA. Gene expression is shown as fold change in mRNA expression relative to that of *GAPDH*. C_P values >35 were considered unreliable. The average and SEM of three independent experiments are shown. *P<0.05, **P<0.01, ***P<0.001.

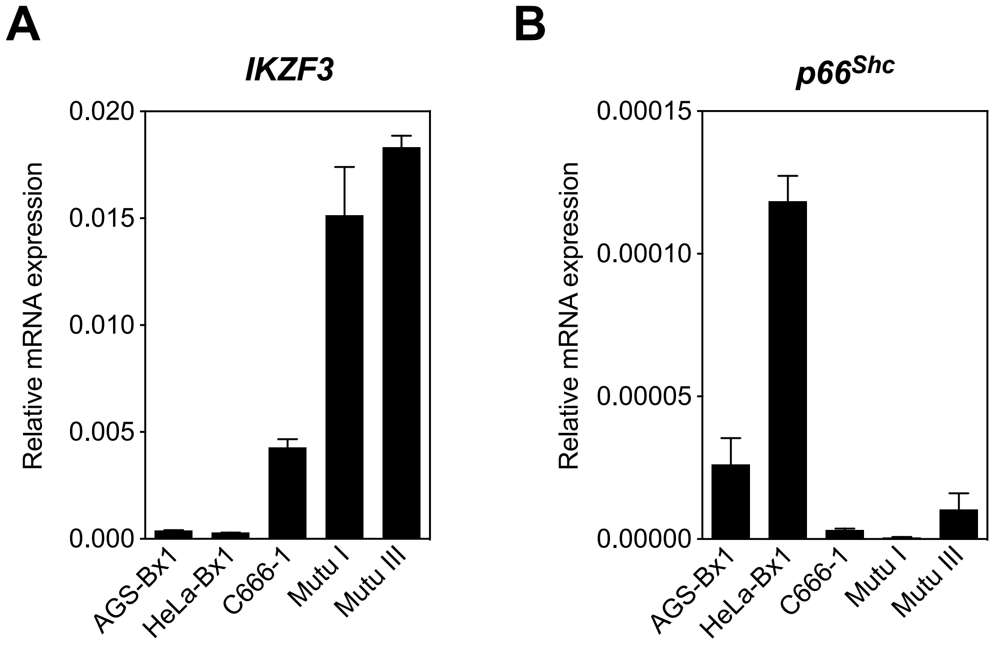

Supplementary figure 3 Relative gene expression of *IKZF3* (A) and *F3* (B *p66*^{Shc} (B) in EBV-positive cell lines AGS-Bx1, HeLa-Bx1, C666-1, Mutu I and Mutu III analyzed by RT-qPCR. Gene expression is shown as mRNA expression relative to that of *ACTB*. The average and SEM of at least two independent experiments are shown.

Supplementary table 1 RNA-seq reads.

A

Sample	Raw reads	Filtered reads	Mapped to human genome
C666-1 Gap-CTL #1	130,320,036	128,896,420	122,357,404
C666-1 Gap-CTL #2	130,811,438	129,743,974	123,449,754
C666-1 Gap-BART #1	133,609,546	132,380,916	126,378,620
C666-1 Gap-BART #2	126,998,364	125,875,392	119,540,534

B

Sample	Unmapped reads	Mapped to EBV genome
C666-1 Gap-CTL #1	6,539,016	384,110
C666-1 Gap-CTL #2	6,002,296	167,628
C666-1 Gap-BART #1	6,294,220	257,428
C666-1 Gap-BART #2	6,334,858	143,578

Supplementary table 2 Sequences of oligos used in this study.

Oligo	Sequence (5'-3')	Reference
V(A/B)-F	TCTTCTAGAGGACGCAGGATATCTGCAGGATCAGAGG	Yamamoto *et al*. 2012(1)
VII(A/B)-R	AGGGGATCCGCACCCCCAGTACCGGGCCATCCG	Yamamoto *et al*. 2012 (1)
V-F	GAAAAGCTTGGGATTAATGCCTGGACCCTCACCAG	Yamamoto *et al*. 2012 (1)
VII(D)-R	AGGGGATCCCCCGCCACCACGGTGCAGCCTAC	Yamamoto *et al*. 2012 (1)
Gap-CTL	A*A*C*A*C*G*T*C*T*A*T*A*C*G*C*	N/A
Gap-BART_exon III	C*G*G*A*C*C*T*G*A*A*A*C*G*A*G*A*	N/A
Gap-BART_exon V	T*C*A*T*T*C*C*A*T*C*G*G*A*T*A*A*	N/A
Gap-BART_exon VII	T*A*C*A*C*C*T*T*C*A*T*C*A*C*G*G*	N/A
BART_ exon III-F	GGGGTTGTAACGGAAGGCAA	N/A
BART_exon III-R	TGTCCGGTAAACGCCATACG	N/A
BART_exon V-F	CGGCCCAGTAGCTTGATGAC	N/A
BART_exon V-R	GGTGTACACGCACGAGAAATG	N/A
BART_exon VII-F	AAGTCAGGAATGCCTTCTGGTG	N/A
BART_exon VII-R	CACACCTGTTTACCCAGACC	N/A
ACTB-F	CACTCTTCCAGCCTTCCTTC	Cao *et al*. 2015 (2)
ACTB-R	GTACAGGTCTTTGCGGATGT	Cao *et al*. 2015 (2)
AIFM3-F	TCCCATTGTGTCCAAGGTCG	N/A
AIFM3-R	AGGACATGTCGCCAGTCTTG	N/A
ALDH1A3-F	ATAAGCCCGACGTGGACAAG	N/A
ALDH1A3-R	GAACCCGGCGGCCAAG	N/A
AOX1-F	TCATCTAAGGGTCTGGGAGAGT	N/A
AOX1-R	TCCAGGTTCATCTCTCGGAA	N/A
CD274-F	TGCAGGGCATTCCAGAAAGA	N/A
CD274-R	TAGGTCCTTGGGAACCGTGA	N/A
CD7-F	CCTGTAGACCCAGAGAGGCT	N/A
CD7-R	GGAGACTGCTGCACCTCTT	N/A
CDH1-F	TGCCCAGAAAATGAAAAAGG	Albino *et al*. 2012 (3)
CDH1-R	GTGTATGTGGCAATGCGTTC	Albino *et al*. 2012 (3)

CDH16-F	TGCAGAGCTGTCTGTGGAAG	Sancisi *et al*. 2013 (4)
CDH16-R	CCCCTGACAGCACGATCT	Sancisi *et al*. 2013 (4)
CDK8-F	CAGAGGCTGTGACAATGGACT	N/A
CDK8-R	ATCCTTCCCATCTTTCCTCTTGG	N/A
CLDN11-F	CTCATCCTGCCGGGCTAC	N/A
CLDN11-R	CGGATGCAGGGAAGAACAGT	N/A
CLDN14-F	TCTTGCACATGGGAGCTGGA	N/A
CLDN14-R	CTTCCAGGCATGCAGAAAACCT	N/A
CLDN18-F	TTCCATCCCAGTACCAAAGC	Ito *et al*. 2011 (5)
CLDN18-R	CCGTTCTTTCCCCAGACATA	Ito *et al*. 2011 (5)
CXCL10-F	TTCAAGGAGTACCTCTCTCTAG	N/A
CXCL10-R	CTGGATTCAGACATCTCTTCTC	N/A
CXCL8-F	TCCTGATTTCTGCAGCTCTGT	N/A
CXCL8-R	AAATTTGGGGTGGAAAGGTT	N/A
CXCR2-F	TGGATTTCCCCCTTGCAACC	N/A
CXCR2-R	AAATCCTGACTGGGTCGCTG	N/A
DDX58-F	GTGCAAAGCCTTGGCATGT	N/A
DDX58-R	TGGCTTGGGATGTGGTCTACTC	N/A
DEFB4A-F	GCTTGATGTCCTCCCCAGACT	N/A
DEFB4A-R	CAGGATCGCCTATACCACCAAA	N/A
DTX3-F	TTCCATGGGCTTTCCAGGTC	N/A
DTX3-R	GCCATTCTGGACAGGACGAA	N/A
ECM1-F	GGTGGGCTGCTCACACATTC	N/A
ECM1-R	AGGGGGACCCACTTCCTTTT	N/A
ELF3-F	CCTGGCGGAACTGGATTTCT	N/A
ELF3-R	CATGAGGCTACCGGAGTGG	N/A
GAPDH-F	GAAGGTGAAGGTCGGAGTA	N/A
GAPDH-R	GAAGATGGTGATGGGATTTC	N/A
GAPLINC-F	TGGACTCAGGCACGTTTACA	N/A
GAPLINC-R	TGGCACAATCAGGGCTCTTG	N/A

GP1BA-F	TGTGAGGTCTCCAAAGTGGC	N/A
GP1BA-R	TAAGGCATCAGGGTTGCCAG	N/A
GP5-F	GCTTGCAAGTGTGTCTTCCG	N/A
GP5-R	GGCTGTCGGAGATCATGAGG	N/A
HSD17B12-F	GGAGCAGCGCCTATTAGTGT	N/A
HSD17B12-R	CGAAATACGCAGGGCTAGGT	N/A
IFIH1-F	GTTGAAAAGGCTGGCTGAAAAC	N/A
IFIH1-R	TCGATAACTCCTGAACCACTG	N/A
IFIT1-F	TAGCCAACATGTCCTCACAGAC	N/A
IFIT1-R	TCTTCTACCACTGGTTTCATGC	N/A
IFIT2-F	GGTCTCTTCAGCATTTATTGGTG	Imaizumi *et al*. 2014 (6)
IFIT2-R	TGCCGTAGGCTGCTCTCCA	Imaizumi *et al*. 2014 (6)
IFNA1-F	CAGTTCCAGAAGGCTCCAGC	Li et *et al*. 2014 (7)
IFNA1-R	TCCTCATCCCAAGCAGCAG	Li et *et al*. 2014 (7)
IFNB1-F	TTGAATGGGAGGCTTGAATA	N/A
IFNB1-R	CTATGGTCCAGGCACAGTGA	N/A
IFNL1-F	CGCCTTGGAAGAGTCACTCA	Bayer *et al*. 2016 (8)
IFNL1-R	GAAGCCTCAGGTCCCAATTC	Bayer *et al*. 2016 (8)
IFNL2-F	ACATAGCCCAGTTCAAGTC	Bayer *et al*. 2016 (8)
IFNL2-R	GACTCTTCTAAGGCATCTTTG	Bayer *et al*. 2016 (8)
IL10-F	CGAGATGCCTTCAGCAGAGT	N/A
IL10-R	CGCCTTGATGTCTGGGTCTT	N/A
IL13-F	TGAGGAGCTGGTCAACATCA	Stellato *et al*. 2011 (9)
IL13-R	CAGGTTGATGCTCCATACCAT	Stellato *et al*. 2011 (9)
IL5-F	GAGACCTTGGCACTGCTTTC	N/A
IL5-R	CCCCTTGCACAGTTTGACTC	N/A
IL6-F	CCCTGAGAAAGGAGACATGTAA	N/A
IL6-R	TCTTTTTCAGCCATCTTTGGA	N/A
IL7R-F	CCAACCGGCAGCAATGTATG	N/A
IL7R-R	AGGATCCATCTCCCCTGAGC	N/A

IKZF3-F	AGAGAAGTTCCCTTGAGGAG	Veistinen *et al*. 2002 (10)
IKZF3-R	TAGCTGATGGCGTTATTGATGG	Veistinen *et al*. 2002 (10)
IRF7-F	GCAGCGTGAGGGTGTGTCTT	Li *et al*. 2011 (11)
IRF7-R	GCTCCATAAGGAAGCACTCGAT	Li *et al*. 2011 (11)
ISG20-F	CTCCACGAACGTCAAATGCG	N/A
ISG20-R	ATCAAGTGAAGTCAGGGGCG	N/A
ITGA10-F	CTGACAGGTCTCTGCTCCCCC	Mertens-Walker *et al*. 2015 (12)
ITGA10-R	CCATCGCTGTCCACCCCCAA	Mertens-Walker *et al*. 2015 (12)
ITGA5-F	CAAAGGGCCTGGAGTTGGAT	N/A
ITGA5-R	AGCCTGAAACACTCAGCCTC	N/A
ITGA9-F	CAAAGGCATCGGCAAGGTTT	N/A
ITGA9-R	TCCCCATTCAGGTCAACTGC	N/A
ITGB7-F	TGCCGTCTCCCAGATCAGTA	N/A
ITGB7-R	GTTTCCACATAGGTGCGTGC	N/A
KCNQ1OT1-F	TACCGGATCCAGGTTTGCAGTACA	Cao *et al*. 2015 (2)
KCNQ1OT1-R	GCTGATAAAGGCACCGGAAGGAAA	Cao *et al*. 2015 (2)
KDM1B-F	ACAGAATTGGAGGCCGAGTC	N/A
KDM1B-R	GAGCGAGCAGATACTTGTTCA	N/A
LPP-F	AGACAAGATCGGCCTGAAGAA	N/A
LPP-R	CCTCTGATGAGCGTCCAGTG	N/A
OAS1-F	CGTTGGTGTTGGCATCTTCT	N/A
OAS1-R	TGTCTGCATTGTCGGCACTT	N/A
OLR1-F	GAGTAGCAAATTGTTCAGCTCCT	N/A
OLR1-R	CTCGGACTCTAAATCAGATCAGC	N/A
ORM1-F	CACCACCTACCTGAATGTCCAGC	Porez et *et al*. 2013 (13)
ORM1-R	GATCAGCAAGTGAGCGAAATGC	Porez et *et al*. 2013 (13)
$p66^{Shc}$-F	AATGAGTCTCTGTCATCGCTGGAG	Li et *et al*. 2014 (14)
$p66^{Shc}$-R	GGCGATGATCTGTTTGCAGTCTGCGG	Li et *et al*. 2014 (14)
PCDHAC2-F	CTGGGGAGCTGATAGCCAGA	N/A
PCDHAC2-R	CCCCTGCTTAACGGGTCTTT	N/A

PDPN-F	CAACAGTGACCCTGGTTGGA	N/A
PDPN-R	GGCGTAACCCTTCAGCTCTT	N/A
SCARF1-F	ACAGTCTCACATCACGACCC	N/A
SCARF1-R	CTTGGGGTGGCACACAGTAG	N/A
SCCPDH-F	AGCTGGGAAGACCAACACTG	N/A
SCCPDH-R	TCAAGCGAGGCTGGATTAGC	N/A
SELE-F	GGCAGTGGACACAGCAAATC	N/A
SELE-R	TGGACAGCATCGCATCTCA	N/A
SEPT9-F	TGAAGAAGTCTTACTCAGGAGGC	N/A
SEPT9-R	TGGAATTTCTGGGTGGAGCTG	N/A
TINAG-F	GTTCCAAGGAGAAGCCCACA	N/A
TINAG-R	CCGGTCCACATTCTCTGGAA	N/A
TLR3-F	TGTCTGGAAGAAAGGGACTTTGA	N/A
TLR3-R	GTTGAACTGCATGATGTACCTTGA	N/A
TNF-F	TCTTCTCCTTCCTGATCGTG	N/A
TNF-R	GCCAGAGGGCTGATTAGAGA	N/A
TNFAIP6-F	AGCACGGTCTGGCAAATACA	N/A
TNFAIP6-R	ATCCATCCAGCAGCACAGAC	N/A
WISP1-F	GTAAGATGTGCGCTCAGCAG	N/A
WISP1-R	GAAGGACTGGCCGTTGTTGT	N/A
oriPtL_BamHI-F	CGGGGATCCCGTTGCACCCACAACATGTAAGGGC	N/A
oriPtL-KpnI-R	GGGGTACCCCTGACACGGGGGGGGACCAAAC	N/A
ChIP_IFNB1_-41-F	AGGACCATCTCATATAAATAGGCCATACCC	Li et *et al*. 2011(15)
ChIP_IFNB1_-41-R	ACTGAAAATTGCTGCTTCTTTGTAGGAATC	Li et *et al*. 2011 (15)
ChIP_IFNB1_+644-F	CTCCTAGCCTGTGCCTCTGG	N/A
ChIP_IFNB1_+644-R	ATGCAGTACATTAGCCATCAGTCA	N/A
ChIP_CXCL8_-49-F	GGGCCATCAGTTGCAAATC	Imamura *et al*. 2014 (16)
ChIP_CXCL8_-49-R	TTCCTTCCGGTGGTTTCTTC	Imamura *et al*. 2014 (16)
ChIP_CXCL8_+2918-F	CCAGCTGTGTTGGTAGTGCT	N/A
ChIP_CXCL8_+2918-R	AAACAAGTTTCAACCAGCAAGAA	N/A

A star indicates that the nucleotide has a phosphorothioate backbone and an undisclosed number of flanking nucleotides are LNA nucleotides. N/A; not applicable.

Supplementary table 2 references

1. **Yamamoto T, Iwatsuki K.** 2012. Diversity of Epstein-Barr virus BamHI-A rightward transcripts and their expression patterns in lytic and latent infections. Journal of medical microbiology **61:**1445-1453.
2. **Cao S, Moss W, O'Grady T, Concha M, Strong MJ, Wang X, Yu Y, Baddoo M, Zhang K, Fewell C, Lin Z, Dong Y, Flemington EK.** 2015. New Noncoding Lytic Transcripts Derived from the Epstein-Barr Virus Latency Origin of Replication, oriP, Are Hyperedited, Bind the Paraspeckle Protein, NONO/p54nrb, and Support Viral Lytic Transcription. J Virol **89:**7120-7132.
3. **Albino D, Longoni N, Curti L, Mello-Grand M, Pinton S, Civenni G, Thalmann G, D'Ambrosio G, Sarti M, Sessa F, Chiorino G, Catapano CV, Carbone GM.** 2012. ESE3/EHF controls epithelial cell differentiation and its loss leads to prostate tumors with mesenchymal and stem-like features. Cancer Res **72:**2889-2900.
4. **Sancisi V, Gandolfi G, Ragazzi M, Nicoli D, Tamagnini I, Piana S, Ciarrocchi A.** 2013. Cadherin 6 is a new RUNX2 target in TGF-beta signalling pathway. PLoS One **8:**e75489.
5. **Ito T, Kojima T, Yamaguchi H, Kyuno D, Kimura Y, Imamura M, Takasawa A, Murata M, Tanaka S, Hirata K, Sawada N.** 2011. Transcriptional regulation of claudin-18 via specific protein kinase C signaling pathways and modification of DNA methylation in human pancreatic cancer cells. J Cell Biochem **112:**1761-1772.
6. **Imaizumi T, Numata A, Yano C, Yoshida H, Meng P, Hayakari R, Xing F, Wang L, Matsumiya T, Tanji K, Tatsuta T, Murakami M, Tanaka H.** 2014. ISG54 and ISG56 are induced by TLR3 signaling in U373MG human astrocytoma cells: possible involvement in CXCL10 expression. Neurosci Res **84:**34-42.
7. **Li YF, Lee KG, Ou X, Lam KP.** 2014. Bruton's tyrosine kinase and protein kinase C micro are required for TLR7/9-induced IKKalpha and IRF-1 activation and interferon-beta production in conventional dendritic cells. PLoS One **9:**e105420.
8. **Bayer A, Lennemann NJ, Ouyang Y, Bramley JC, Morosky S, Marques ET, Jr., Cherry S, Sadovsky Y, Coyne CB.** 2016. Type III Interferons Produced by Human Placental Trophoblasts Confer Protection against Zika Virus Infection. Cell Host Microbe **19:**705-712.
9. **Stellato C, Gubin MM, Magee JD, Fang X, Fan J, Tartar DM, Chen J, Dahm GM, Calaluce R, Mori F, Jackson GA, Casolaro V, Franklin CL, Atasoy U.** 2011. Coordinate regulation of GATA-3 and Th2 cytokine gene expression by the RNA-binding protein HuR. J Immunol **187:**441-449.

10. **Veistinen E, Liippo J, Lassila O.** 2002. Quantification of human Aiolos splice variants by real-time PCR. J Immunol Methods **271:**113-123.

11. **Li Q, Tainsky MA.** 2011. Epigenetic silencing of IRF7 and/or IRF5 in lung cancer cells leads to increased sensitivity to oncolytic viruses. PLoS One **6:**e28683.

12. **Mertens-Walker I, Fernandini BC, Maharaj MS, Rockstroh A, Nelson CC, Herington AC, Stephenson SA.** 2015. The tumour-promoting receptor tyrosine kinase, EphB4, regulates expression of integrin-beta8 in prostate cancer cells. BMC Cancer **15:**164.

13. **Porez G, Gross B, Prawitt J, Gheeraert C, Berrabah W, Alexandre J, Staels B, Lefebvre P.** 2013. The hepatic orosomucoid/alpha1-acid glycoprotein gene cluster is regulated by the nuclear bile acid receptor FXR. Endocrinology **154:**3690-3701.

14. **Li X, Xu Z, Du W, Zhang Z, Wei Y, Wang H, Zhu Z, Qin L, Wang L, Niu Q, Zhao X, Girard L, Gong Y, Ma Z, Sun B, Yao Z, Minna JD, Terada LS, Liu Z.** 2014. Aiolos promotes anchorage independence by silencing p66Shc transcription in cancer cells. Cancer Cell **25:**575-589.

15. **Li P, Wong JJ, Sum C, Sin WX, Ng KQ, Koh MB, Chin KC.** 2011. IRF8 and IRF3 cooperatively regulate rapid interferon-beta induction in human blood monocytes. Blood **117:**2847-2854.

16. **Imamura K, Imamachi N, Akizuki G, Kumakura M, Kawaguchi A, Nagata K, Kato A, Kawaguchi Y, Sato H, Yoneda M, Kai C, Yada T, Suzuki Y, Yamada T, Ozawa T, Kaneki K, Inoue T, Kobayashi M, Kodama T, Wada Y, Sekimizu K, Akimitsu N.** 2014. Long noncoding RNA NEAT1-dependent SFPQ relocation from promoter region to paraspeckle mediates IL8 expression upon immune stimuli. Mol Cell **53:**393-406.
